# Rituximab in systemic sclerosis: A protocol for systematic review

**DOI:** 10.1097/MD.0000000000017110

**Published:** 2019-09-20

**Authors:** Marina Maria Vieira de Figueiredo Caldas, Francisco Alves Bezerra Neto, Kesley Pablo Morais de Azevedo, Isac Davidson Santiago Fernandes Pimenta, Ana Katherine Da Silveira Gonçalves De Oliveira, Grasiela Piuvezam

**Affiliations:** a Federal University of Rio Grande do Norte; b Department of Clinical Medicine; c Post-Graduation Program in Public Health (PPGSCol); d Post-Graduation Program in Public Health (PPGSCol), Federal University of Rio Grande do Norte, Rio Grande do Norte; e Department of Tocoginecology, Post-Graduation Program in Health Sciences; f Department of Public Health, Post-Graduation Program in Public Health (PPGSCol), Federal University of Rio Grande do Norte, Rio Grande do Norte, Brazil.

**Keywords:** cutaneous fibrosis, interstitial lung disease, rituximab, systematic review, systemic sclerosis

## Abstract

**Background:**

Systemic sclerosis (SSc) is a clinically complex and challenging disease, the most frequent complication of which is interstitial lung disease, which leads to a worse prognosis. In this situation, cyclophosphamide is considered the criterion standard for treatment, despite the controversies regarding its efficacy and toxicity. However, studies using rituximab (RTX) have shown that this drug may be a promising therapeutic option. The objective is to describe a protocol of a systematic review (SR) that analyzes the scientific evidence on the effects of RTX on SSc.

**Methods:**

This protocol is guided by the Preferred Reporting Items for Systematic Reviews and Meta-Analyses Protocols. The databases to be searched are PubMed, Scopus, SciELO, LILACS, ScienceDirect, Web of Science, COCHRANE, WHOLIS, PAHO, and EMBASE. The studies that would be included in SR are clinical trials that evaluate the use of RTX in patients with SSc who meet the classification criteria for the disease according to American College of Rheumatology and European League Against Rheumatism (2013) and/or LeRoy criteria will be included in the SR. The data to be extracted are related to the characteristics of the studies: authors, year of publication, study location, type of study, sample size and age, patient characteristics, duration of intervention, therapeutic scheme, follow-up time, main variables, and main results.

**Results:**

In our study, we hope to find articles presenting new evidence supporting treatment of SSc with RTX.

**Conclusions:**

The SR will present results of scientific evidence for the effects of RTX in SSc. We hope that the results could strengthen clinical decisions for the best treatment of SSc and guide future researches.

**PROSPERO registration number:**

CRD42019132018.

## 1 Introduction

Systemic sclerosis (SSc) is an autoimmune connective tissue disease characterized by vascular dysfunction and excessive collagen deposition, resulting in skin fibrosis and the involvement of internal organs.^[1–4]^ SSc affects about 2 per 10,000 individuals and women are more affected than men.^[2,4]^ The most affected age group is between 45 and 64 years of age, and African-Americans have been reported to have an earlier onset and more severe course.^[1,3]^

The pathogenesis of SSc is not well-understood, but B cell abnormalities (autoantibody production, hypergammaglobulinemia, and polyclonal B cell hyperactivity) are part of this complex disorder.^[5,6]^ The disease is associated with significant incapacity and mortality.^[2]^ Pulmonary involvement is a common clinical presentation, and interstitial lung disease (ILD) and pulmonary artery hypertension are the 2 major causes of death in SSc.^[4,7–9]^

SSc can be divided into 2 main subsets, according to the degree of skin involvement. In the limited form, skin thickening is confined to the elbows and knees and internal organ involvement is less severe. The diffuse form is characterized by skin thickening in both proximal and distal to the elbows and knees and/or the trunk, and organ damage is more severe.^[1,2,10,11]^

Treatment for SSc-associated ILD is based on the European League Against Rheumatism (EULAR) recommendations, that is, the use of cyclophosphamide (CYC).^[2,8,12,13]^ This drug is, however, associated with teratogenicity, gonadal failure, bone marrow suppression, and infection.^[2]^ Recently, other immune-based, targeted therapies have been investigated. Hematopoietic stem cell transplantation and B cell depletion therapy (CD20) have shown good results.^[12,14,15]^ In addition, rituximab (RTX) is a monoclonal chimeric antibody against CD20 that depletes peripheral B cells. It is used in systemic rheumatic diseases and its use in SSc has been proposed because of the growing evidence for the role of B cells in SSc.^[16]^

The incapacity and mortality caused by SSc-ILD, the fragility of current therapies, and the new evidence supporting treatment with RTX justify the importance of developing a protocol to systematic review (SR) that analyzes the scientific evidence on the effects of RTX on SSc.

The objective of this study is to describe a protocol of an SR that analyzes the scientific evidence on the effects of RTX on SSc to support clinical decisions for the best treatment of SSc.

## 2 Methods

### 2.1 Protocol and registration

This protocol was prepared in accordance with the guidelines described by the Preferred Reporting Items for Systematic Reviews and Meta-Analyses Protocols (PRISMA-P).^[17]^ Posteriorly, the SR will be prepered in accordance with the guidelines of Preferred Reporting Items for Systematic Reviews and Meta-Analyses (PRISMA).^[18]^

The protocol was registered with the International Prospective Register of Systematic Reviews (PROSPERO) on 24 July 2019 (CRD42019132018).

### 2.2 Eligibility criteria

#### 2.2.1 Inclusion criteria

Articles are going to be included in the SR if they are clinical trials evaluating the use of RTX in patients with SSc, using the ACR/EULAR (2013) and/or LeRoy classification criteria for SSc.

#### 2.2.2 Exclusion criteria

Reviews, case reports, abstracts, thesis, and other types of epidemiologic studies will be excluded of the SR.

### 2.3 Information sources and literature search

The search will be performed independently by the researchers MMVFC, KPMA, and IDSFP in the following databases: PUBMED, SCOPUS, SCIELO, LILACS, SCIENCE DIRECT, WEB OF SCIENCE, COCHRANE, WHOLIS, PAHO, and EMBASE. Combinations between the keywords “rituximab,” “scleroderma systemic,” and “systemic sclerosis” will be used to guarantee a broad research strategy, according to the characteristics of each database, accompanied by the Boolean operators “AND” and “OR”. The following search strategy will be used: [(RITUXIMAB) AND (SCLERODERMA SYSTEMIC OR SYSTEMIC SCLEROSIS)].

**Figure 1:**
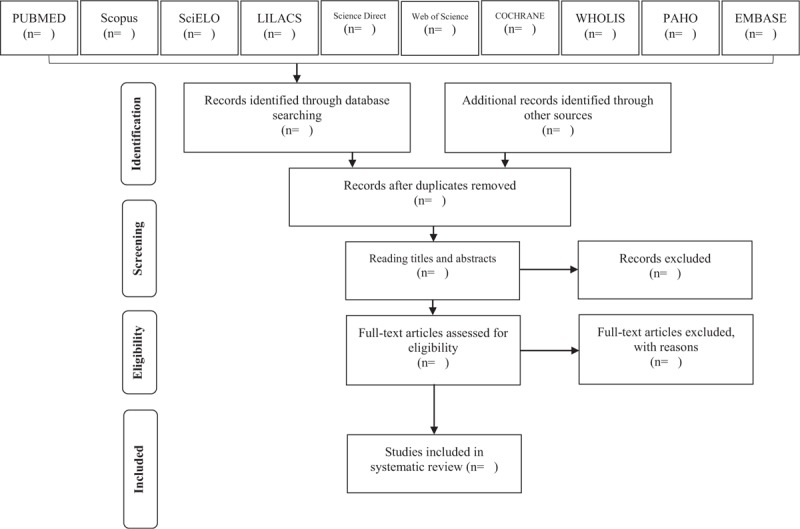
Article selection flowchart. Adapted from Preferred Reporting Items for Systematic Reviews and Meta-Analyses Protocols (PRISMA-P).^[18]^

The first selection will be focused on the title and abstract, with no limitations on the publication date. In this stage, all articles that do not directly address the subject of interest will be excluded, and, using Mendeley software, duplicated titles will be removed (Fig. 1).

The articles that meet the criteria will be directed to a full reading (second stage). After reading the complete articles, both researchers will select the articles to be included in the review. The evaluations will be performed by 2 authors (MMVFC and FABN) and the discrepancies or doubts will be resolved under the guidance of a third researcher (GP).

### 2.4 Data extraction

The following data will be extracted from the selected articles: authors, year of publication, study location, type of study, sample size and age, patient characteristics, duration of intervention, therapeutic scheme, follow-up time, main variables, and main results.

Two reviewers will be responsible for extracting and managing the data, which will be inserted into an EXCEL spreadsheet; doubts will be clarified with the help of the third researcher.

### 2.5 Risk of bias assessment and grading of recommendations assessment, development, and evaluation assessment

The PEDro scale will be used to evaluate the quality of the selected articles.^[19]^ This scale has been validated and is used in randomized clinical trials. It has 11 items, but only 10 (items 2–11) are related to internal validity. According to the evaluation of the established criteria, the articles can obtain 0 (does not meet) or 1 (meet) point for each item evaluated. The sum of the scores varies from 0 to 10. The evaluations will be performed by 2 authors and discrepancies or doubts will be resolved under the guidance of a third researcher.

### 2.6 Data analysis and synthesis

The SR will present information from the included studies, such as the sample characteristics, types of measurements and assessments, primary outcomes (improvement of dyspnea and quality of life), and secondary outcomes (improvement of skin fibrosis and evaluation of adverse events), statistical analysis, and the main results.

A narrative approach will be used to summarize the effectiveness of the interventions. The heterogeneity between trial results will be evaluated using a standard *I*^2^ test with a significance level of 0.1 and the *I*^2^ statistic, which is a quantitative measure of inconsistency across studies, with a value of 0% indicating no observed heterogeneity, and values of 50% indicating substantial levels are present. If there is heterogeneity (*I*^2^ = 75%), a random-effects model will be used to combine the trials to calculate the relative risk and 95% confidence interval, using the DerSimonian-Laird algorithm in meta for package, a meta-analysis package for R. Other study characteristics and results will be summarized narratively if a meta-analysis cannot be performed for all or some of the included studies. If possible, funnel plots will also be used to assess the presence of potential reporting bias, and a linear regression approach will be used to evaluate funnel plot asymmetry.

For analysis of subgroups, the types of intervention will be with the use of RTX vs control; sex, age group, and clinical conditions will be considered.

## 3 Discussion

The SR proposal presented in this protocol aims to identify studies that show the scientific evidence for the effects of RTX in SSc, particularly regarding lung and skin involvement. Studies have demonstrated the role of B lymphocytes in the pathophysiology of SSc and associated fibrosis.^[5,6]^ Moreover, studies using RTX, a monoclonal chimeric antibody that depletes peripheral B cells, have shown that this drug may be a promising therapeutic option with the possibility that only 1 drug may be beneficial to both the skin and lung in this disease.^[12,15,16]^

Currently, many studies have demonstrated a possible role for RTX in the management of patients with SSc. Some studies suggested that RTX has a beneficial effect on lung function and skin fibrosis in patients with SSc.^[12,15,16,20,21]^ Others, have, showed inconclusive data about the efficacy of RTX in patients with SSc.^[22]^ The main adverse events associated with the use of RTX in SSc were mild infusion reactions, besides sepsis, urinary tract, pulmonary, herpes zoster, and cardiovascular involvement.^[12,15,21,23]^

Following EULAR the recommendations, 2 studies guided the treatment of ILD associated with SSc with CYC.^[13]^ Both studies considered forced vital capacity (FVC) analysis as the primary outcome and computed tomography (CT) lung analysis as a secondary outcome. Tashkin et al^[24]^ reported that 1 year of oral CYC treatment resulted in a slight but significant improvement in FVC and total lung capacity. Conversely, Hoyles et al^[25]^ found no significant improvement in FVC and CT in the CYC group.

According to the EULAR recommendations for cutaneous fibrosis in SSc, methotrexate (MTX) is indicated as the criterion standard for treatment. The EULAR MTX treatment protocol was based on 2 studies indicating that this drug improves the modified Rodnan skin score, but the effects on other organs have not been established. The study by van den Hoogen et al^[26]^ concluded that a greater number of SSc patients responded favorably to MTX compared to placebo. The results of Pope et al^[27]^ showed a favorable trend with the use of MTX over placebo, but the differences between groups were considered subtle.^[27]^ However, despite the EULAR recommendations for the use of MTX, it is important to point out that, from the point of view of patient safety, the use of this drug can cause liver toxicity, pancytopenia, teratogenesis, and lung injury.^[28]^

The literature shows that RTX is used in treatment for many diseases, such as lymphoma and rheumatoid arthritis (RA). The use of RTX for the treatment of non-Hodgkin lymphoma leads to common adverse effects related to infusion, including pruritus, nausea, vomiting, dizziness, headache, fever, and stiffness.^[29]^ These symptoms are usually related to the first infusion of RTX. However, severe infusion reactions occur in approximately 10% of patients. The vast majority of cases with adverse events are reversible with the interruption or discontinuation of RTX in addition to supportive care.^[30]^

The use of RTX in the treatment of RA is associated with adverse effects through infusion reactions, which are mainly graded from mild to moderate; severe reactions are uncommon (<1%).^[31]^ Regarding the risk of malignancy, no elevations in solid tumor or lymphoma rates have been observed in patients using RTX, except for patients with T cell deficiency in HIV infection.^[31]^

However, there was a need to look for evidence in the literature that brings studies the effects of RTX in SSc. Therefore the proposed SR may provide important information for better treatment for patients with SSc and also help to highlight areas that require the need for further research on the subject.

## Author contributions

**Conceptualization:** Marina Maria Vieira de Figueiredo Caldas, Francisco Alves Bezerra Neto, Grasiela Piuvezam.

**Formal analysis:** Francisco Alves Bezerra Neto, Kesley Pablo Morais de Azevedo, Ana Katherine da Silveira Gonçalves de Oliveira, Grasiela Piuvezam.

**Investigation:** Marina Maria Vieira de Figueiredo Caldas, Francisco Alves Bezerra Neto, Kesley Pablo Morais de Azevedo, Isac Davidson Santiago Fernandes Pimenta, Grasiela Piuvezam.

**Methodology:** Francisco Alves Bezerra Neto, Kesley Pablo Morais de Azevedo, Ana Katherine da Silveira Gonçalves de Oliveira, Grasiela Piuvezam.

**Project administration:** Francisco Alves Bezerra Neto, Kesley Pablo Morais de Azevedo, Grasiela Piuvezam.

**Resources:** Marina Maria Vieira de Figueiredo Caldas, Francisco Alves Bezerra Neto, Isac Davidson Santiago Fernandes Pimenta.

**Software:** Isac Davidson Santiago Fernandes Pimenta.

**Supervision:** Francisco Alves Bezerra Neto, Grasiela Piuvezam.

**Writing – original draft:** Marina Maria Vieira de Figueiredo Caldas, Francisco Alves Bezerra Neto, Kesley Pablo Morais de Azevedo, Grasiela Piuvezam.

**Writing – review & editing:** Marina Maria Vieira de Figueiredo Caldas, Francisco Alves Bezerra Neto, Kesley Pablo Morais de Azevedo, Ana Katherine da Silveira Gonçalves de Oliveira, Grasiela Piuvezam.
